# Considerations for designing trials targeting muscle dysfunction in exercise oncology

**DOI:** 10.3389/fphys.2023.1120223

**Published:** 2023-02-09

**Authors:** Alexander Brooks, Alec Schumpp, Jake Dawson, Emily Andriello, Ciaran Michael Fairman

**Affiliations:** Exercise Oncology Laboratory, University of SC, Exercise Science, Columbia, SC, United States

**Keywords:** muscle mass, muscle strength, cachexia, sarcopenia, frailty

## Abstract

Individuals diagnosed with cancer commonly experience a significant decline in muscle mass and physical function collectively referred to as cancer related muscle dysfunction. This is concerning because impairments in functional capacity are associated with an increased risk for the development of disability and subsequent mortality. Notably, exercise offers a potential intervention to combat cancer related muscle dysfunction. Despite this, research is limited on the efficacy of exercise when implemented in such a population. Thus, the purpose of this mini review is to offer critical considerations for researchers seeking to design studies pertaining to cancer related muscle dysfunction. Namely, 1) defining the condition of interest, 2) determining the most appropriate outcome and methods of assessment, 3) establishing the best timepoint (along the cancer continuum) to intervene, and 4) understanding how exercise prescription can be configured to optimize outcomes.

## Introduction

Individuals with cancer commonly experience a significant decline in muscle mass and physical function. So much so, that cancer is widely accepted as an “accelerated aging process” in which declines in muscle mass and function that would typically take decades occur in only months (Christensen et al., 2014; Guida et al., 2019; Wang et al., 2021). Concerningly, declines in muscle mass during treatment are associated with a higher incidence of chemotherapy toxicity, poorer surgical outcomes, physical impairment, and increased risk of mortality (Aversa et al., 2017; Au et al., 2021). The intersection between aging and oncology has resulted in an ever-increasing number of individuals experiencing a loss of skeletal muscle mass and function, highlighting the need for the development of therapeutic approaches to combat cancer related muscle dysfunction.

Exercise is rapidly emerging as a well-tolerated and safe therapy that has potential to improve muscle mass and physical function in individuals with cancer (Segal et al., 2017; Campbell et al., 2019). However, numerous challenges exist when attempting to prevent muscle loss with exercise in clinical settings, such as the impact of tumor-/treatment-related factors, presence of comorbidities, injuries, disease progression, bone metastases, and concomitant medications (Aversa et al., 2017; Conte et al., 2020; Fairman et al., 2022). Importantly, there is a gap in understanding the feasibility and/or effectiveness of exercise interventions in individuals with specific impairments in muscle mass/function who are at the highest risk of further debilitation and intuitively, the greatest need of intervention.

The purpose of this review is to provide considerations for researchers seeking to design studies investigating cancer-related muscle dysfunction. Namely, key considerations in understanding how exercise can be used to manage muscle dysfunction are 1) defining the condition of interest, 2) determining the most appropriate outcome, 3) establishing the best timepoint to intervene, and 4) understanding how exercise prescription can be configured to optimize outcomes.

### What is the operational definition of a given condition?

Muscle dysfunction, herein defined as a measurable impairment in muscular strength and/or composition, is a hallmark impairment in cancer that is estimated to affect 5%–89% of individuals depending on the disease and treatment type (Christensen et al., 2014; Rier et al., 2016). Notably, much of the range in prevalence may be due to discrepancies in methodologies used to assess muscle dysfunction, as well as a lack of consensus regarding definitions. Namely, impairments in muscle mass and/or strength are commonly used as a criterion to diagnose distinct, yet overlapping, conditions such as sarcopenia, frailty, and cachexia (Christensen et al., 2014). Importantly, there is no consensus for the definition, diagnoses, and cutoffs for these conditions. The lack of consistency in operational definitions leads to uncertainty and under/over-diagnosis within conditions, as well as the potential for overlap between conditions. Therefore, depending on which operational definition is used, it is possible to have both discrepancies of prevalence within conditions and overlap between conditions (Prado et al., 2021).

Sarcopenia has traditionally been defined as an age-related decline in muscle mass (Evans and Campbell, 1993; Joseph Melton et al., 2000). Despite this, the development of sarcopenia is now recognized to begin earlier in life and has many contributing factors beyond aging such as genetics, lifestyle, and disease (Cruz-Jentoft et al., 2019). For example, long term conditions including cardiovascular, metabolic, and/or musculoskeletal impairments have been found to increase the probably of sarcopenia, particularly when multiple conditions are present (Dodds et al., 2020). As such, research suggests that both cancer and its treatment accelerate declines towards a sarcopenic condition (Williams et al., 2019). Importantly, sarcopenia is now considered a condition with low muscle strength overtaking the role of low muscle mass as a primary criterion (Schaap et al., 2018; Cruz-Jentoft et al., 2019). Guidelines for assessment of sarcopenia typically incorporate dual-energy x-ray absorptiometry (DXA), bioelectrical impedance analysis (BIA), magnetic resonance imaging (MRI) or computed tomography (CT) (Cruz-Jentoft et al., 2019).

While published guidelines offer multiple assessment methods and cutoffs, it has been demonstrated that agreement between methods of assessment is poor. For example, in a 2021 analysis of 131 individuals scheduled for surgery for gastrointestinal tumors, the prevalence of sarcopenia from DXA derived values ranged from 11.5% to 19.1% whereas when using CT, the prevalence ranged from 3.8% to 26.7% (Simonsen et al., 2021). In addition to variations based on the assessment method, there is also a lack of agreement on cut-off points for diagnosis of sarcopenia. In a 2018 analysis performed by Simonsen and colleagues, it was determined that 22 different cut-off values were applied across 29 studies exploring the clinical implications of sarcopenia (Simonsen et al., 2018). This resulted in a large between-study variation in the prevalence of sarcopenia, ranging from 12% to 78%.

Like sarcopenia, there is disagreement pertaining to diagnostic criteria for frailty, a condition defined as a state of increased vulnerability resulting from aging associated declines across multiple physiological systems (Xue, 2011; Hogan, 2018). For example, an analysis by Bouillon and colleagues identified 27 independent scales to diagnose frailty (Bouillon et al., 2013). The number and type of items (physical function, disease, cognition, nutrition, etc…) included in the frailty instruments varied significantly, and reliability and validity were found to have only been examined in 26% of the instruments. Among the 27 tests identified, two emerged as the most utilized, the phenotype of frailty (69%) and the frailty index (12%).

The phenotype of frailty defines frailty as a physical condition meeting three of five phenotypic criteria including low grip strength, low energy, slowed waking speed, low physical activity, and unintentional weight loss (Fried et al., 2001). Notably, cut off points for the criteria that comprise the frailty phenotype have not been operationalized consistently (Theou and Rockwood, 2015). This has resulted in large variations in prevalence depending on the cut off points that are used. For example, a study of assisted living residents comparing two previously utilized sets of cut off points resulted in a prevalence ranging from 19.2% to 48% depending on the criteria used (Freiheit et al., 2011).

Alternatively, the frailty index is the ratio of health deficits (which can be symptoms, signs, diseases, disabilities, and/or laboratory abnormalities) present in an individual to the total number of health deficits considered (Mitnitski et al., 2001). For example, if 10 of 40 possible deficits were present, the person’s frailty index would be 10/40 or 0.25 (Hogan, 2018). Notably, cut points along the frailty index are not universally accepted. For example, previous research has used cut-points of 0.21, 0.25 and 0.35 for the diagnosis of frailty which resulted in various rates of prevalence (Rockwood et al., 2007; Kulminski et al., 2008; Song et al., 2010; Rockwood et al., 2011).

Depending on the method used to diagnose frailty, the likelihood of exercise being able to reverse a frailty condition may also differ. For example, if the diagnosis of frailty is reliant on low strength, weight loss, physical inactivity, and gait speed, then exercise could potentially target all these components. However, the potential of exercise to reverse frailty in a more comprehensive model incorporating diseases and comorbidities (i.e., the frailty index) may be more challenging.

Potentially the most concerning of the muscle wasting conditions, cancer cachexia is a multifactorial and often irreversible syndrome affecting 50%–80% of individuals (Argilés et al., 2014; Lim et al., 2020). Cancer cachexia is associated with reduced physical function, reduced tolerance to anticancer therapy and reduced survival, with estimates suggesting that it contributes to 20%–30% of cancer related deaths (Fearon et al., 2006; Bachmann et al., 2008; Argilés et al., 2014; Lim et al., 2020). Operational definitions have previously included >5% unintentional weight loss in the past 6 months, 2%–5% weight loss with a body mass index of <20 kg/m^2^, or appendicular skeletal muscle index consistent with sarcopenia (males <7·26 kg/m2; females <5·45 kg/m2)* and weight loss >2% (Fearon et al., 2011). Given that no standard definition of cachexia is available, the prevalence of the condition varies widely based on the criteria that are used for diagnosis. For example, Fox and colleagues compared the proportion of individuals who would be diagnosed with cachexia based on four commonly utilized methods (Fox et al., 2009). Of 8541 patients observed, prevalence ranged from 2.4% to 14.7% based on the method that was used.

In addition to variations in definition, working groups have recommended cachexia be categorized into distinct phases; pre-cachexia, cachexia, and refractory cachexia based on the condition’s severity (Fearon et al., 2011). As such, the classification of stages indicates that the 1) severity of the condition varies and 2) potential therapeutic effect of an intervention, may vary across stages. Due to the lack of a clear definition as well as the variations in severity, the ability to investigate the overall safety and effectiveness of an exercise intervention is limited. As such, the American Society of Clinical Oncology has recognized varied definitions as one of the primary limitations of clinical research in cancer cachexia (Roeland et al., 2020). For instance, exercise may prove to be effective in an individual presenting with pre-cachexia, whereas it may be futile in the case of refractory cachexia.

Overall, the lack of consensus pertaining to the definition and diagnostic criteria of muscle wasting conditions has made it difficult to investigate possible therapeutic approaches. Additionally, though there is considerable overlap in the definitions of frailty, sarcopenia, and cachexia, the differing mechanisms and severity preclude the transfer of results in one condition to be directly applied to another (i.e., successful interventions in a pre-frail condition may not transfer to refractory cachexia) (Table 1). The importance of accurately diagnosing the condition lies in understanding its etiology to implement an appropriate intervention. If we aim to improve our understanding of the feasibility and/or effectiveness of exercise for conditions of muscle dysfunction, having a clear definition and diagnostic criteria of the condition is a mandatory pre-requisite of any intervention. Further, using these criteria as eligibility criteria for entry into a trial should be strongly considered to ensure the participants represent individuals with that condition.

**TABLE 1:** Similarities and differences in sarcopenia, frailty, and cachexia. ✓ = usually present, ? = not necessarily present. Adapted from (Prado et al., 2021).

Criteria	Sarcopenia	Frailty	Cachexia
Weight Loss	?	✓	✓
Low Body Mass Index	?	✓	✓
Low Muscle Mass	✓	✓	✓
Low Muscle Function	✓	✓	✓
Inflammation	?	?	✓
Loss of Appetite	?	?	✓
Low Food Intake	?	?	✓

### What is considered a successful outcome?

There is much discussion regarding what constitutes “success” in cancer research concerning muscle loss. In the context of muscle wasting conditions, the United States Food and Drug Administration (FDA) has traditionally required a dual endpoint of changes in muscle mass and physical function to deem interventions successful (Sadeghi et al., 2018; Hilmi et al., 2019). Despite this, a dual endpoint (particularly one requiring an increase in muscle mass) may not reflect the true utility of an exercise intervention.

Research in individuals with cancer suggests that exercise can result in clinically significant improvements in physical function, whereas the results for changes in muscle mass have been more divergent (Campbell et al., 2019). Further, several studies have reported that while exercise may not result in increased muscle mass, it may attenuate reductions (Halle et al., 2020). Given the challenges of increasing muscle mass in oncology, preservation of muscle mass along with improvements in muscle strength/function may be viewed as a positive outcome. For instance, in a prospective study of older adults, muscular strength, not muscle mass, was found to be associated with reduced mortality (Newman et al., 2006). Similarly, the results of cross-sectional analysis of 3493 older adults indicated that participants with low muscle function were at greater odds for losing physical independence compared to those with low muscle mass (Dos Santos et al., 2017). Resultantly, relying on dual outcomes of muscle mass and function may ultimately result in effective interventions not being translated into clinical practice. This is reflected by the FDA’s recent adoption of a composite end point including quality of life as a primary end point in muscle wasting trials (Roeland et al., 2020; Lambert, 2021).

Overall, future research should aim to elucidate clearer endpoints as far as what constitutes “success” in cancer related muscle dysfunction interventions. Solely relying on a dual endpoint of improvements in muscle mass and function may result in interventions incorrectly being considered ineffective, particularly those that result in improvements in physical function and/or quality of life (Lambert, 2021).

### When should an intervention be implemented to optimize outcomes?

Although exercise guidelines exist for a variety of cancer-related side effects, there is a lack of research pertaining to the optimal timing of an intervention along the cancer continuum and with respect to the severity of muscle wasting (Dennett et al., 2020). The ideal scenario would to be to identify individuals at risk of muscle dysfunction and/or intervene as early as possible. However, early identification of muscle wasting conditions, and appropriate interventions, may not always be possible. As such, a critical consideration for exercise interventions targeting muscle dysfunction in cancer is the timing of an intervention.

Exercise “preconditioning” refers to a practice in which exercise is performed prior to initiating a treatment regimen to prevent declines into a clinically compromised state (Stout et al., 2020). Preliminary evidence has suggested that this practice may have therapeutic value. For example, the results of a recent meta-analysis indicated that prehabilitation for individuals with cancer preparing for surgery resulted in a significant improvement in the 6-min walk test (Michael et al., 2021). However, only one study included in the analysis included individuals who were screened at baseline for a muscle dysfunction condition (Carli et al., 2020). In this study, Carli and colleagues examined the effects of an exercise intervention on postoperative complications in patients with frailty undergoing colorectal cancer resection (Carli et al., 2020). Results indicated that 4–5 weeks of pre-habilitation did not influence postoperative outcomes or frailty status. Though the lack of effect may have been a result of the short intervention duration, it is also important to consider that even if exercise preconditioning is theoretically effective, it may not be feasible. There are several challenges when attempting to intervene prior to treatment such as the emotional, financial and time burden of a recent diagnosis. This, coupled with potentially short time to treatment (particularly in cases where the disease is more aggressive/advanced), can impact the potential positive effects of exercise in these settings. As such, future work should aim to identify the feasibility and potential challenges of exercise interventions in the pre-treatment period for muscle dysfunction.

Another critical point of intervention is during the active treatment period, where loss of muscle may be most pronounced (Pin et al., 2018; Davis and Panikkar, 2019). However, the potential benefit of exercise on muscle mass/function during treatment is likely to vary widely based on tumor biology and treatment burden (Clifford et al., 2021; Fairman et al., 2022). For example, an exercise intervention in men undergoing androgen deprivation therapy for prostate cancer was sufficient to preserve and, in some cases, enhance strength and physical function (Newton et al., 2020). Contrastingly, a study by Capozzi and colleagues found that an exercise intervention in individuals with head and neck cancer receiving treatment was unsuccessful in preventing declines in lean body mass and physical function (Capozzi et al., 2016). Notably, approximately 40% of participants dropped out of the intervention during treatment, and the average attendance was less than 50%. Resultantly, the authors suggested that that certain disease types and/or treatment regimens may present a scenario in which it is not feasible to implement exercise during treatment.

The findings of Capozzi and colleagues support the notion that, paradoxically, individuals who have the highest risk of muscle loss may have tumor types/treatment schedules that make it more difficult to adhere and respond to an intervention. Namely, the two scenarios presented above are significantly different, where “success” in one patient population with comparatively mild muscle loss from cancer treatment, cannot be transferred to another with intensive treatment burden, systemic inflammation, and aggressive muscle loss.

While the timing of an exercise intervention is typically considered with respects to the cancer continuum, the severity of dysfunction should be also be considered to better understand the extent to which (if any) positive adaptations in muscle can be achieved with exercise. For example, in the cases of pre-frailty or pre-cachexia, the loss of muscle may not be the priority in terms of impairments. In these cases, cardiotoxicity or comorbidities may require the most immediate attention. Contrastingly, as the severity of muscle dysfunction worsens, an immediate and comprehensive intervention would be indicated. However, this is complicated by additional challenges to overcoming the burden of the condition. Overall, investigating the optimal time to intervene for the prevention and mitigation of muscle dysfunction in cancer is understudied. Future research that includes an accurate diagnosis of muscle dysfunction in addition to considering the etiology of the disease and treatment burden will provide insight into the feasibility, challenges, and potential benefits of utilizing exercise to combat cancer related muscle dysfunction.

### How can exercise prescription be manipulated to promote positive adaptations?

The American College of Sports Medicine recommendation for improving physical function is that resistance training should be performed 2–3 days per week at an intensity of 60%–75% of 1RM for two to three sets of 8–12 repetitions targeting each of the major muscle groups (Campbell et al., 2019). Based on this recommendation, there is little flexibility to alter volume and intensity over time which may undermine the ability to apply progressive overload and individualization. In severe conditions such as cachexia, it is unclear what volume of exercise is tolerable or safe, let alone sufficient to promote positive adaptation. Malnutrition is also a common concern in certain cancer conditions, particularly cachexia (Gaafer and Zimmers, 2021). Malnutrition coupled with treatment burden, escalating weight loss and accumulating fatigue likely impacts an individual’s ability to tolerate the stress of exercise, recover appropriately, and fuel sufficiently to see positive changes in muscle mass and/or function (Meza-Valderrama et al., 2021). Consequently, more careful design and manipulation of exercise volume, intensity and/or frequency may be required to determine the appropriate intervention in these types of settings. There are several dose-response trials of exercise in various cancer settings ongoing or completed (Brown et al., 2018; Scott et al., 2020). However, none to date have specifically examined different doses of exercise aimed at improving muscle mass or function in individuals with muscle dysfunction.

Ultimately, the design of exercise interventions in oncology should be governed by four principles, individualization, specificity, progressive overload, and rest/recovery. This is especially relevant in a cancer population is which treatment is associated with numerous physiological and psychological side effects that are known to fluctuate in severity throughout the course of treatment and survivorship (Schwartz, 2000; Jim et al., 2011). It is likely that an additional manipulation of training variables such as volume, intensity, and frequency are necessary to optimize adaptations (Fairman et al., 2017).

Overall, resistance exercise offers the potential for a therapeutic approach to combat muscle dysfunction. However, more research is needed to better understand the minimal effective dose of exercise required to illicit positive adaptations, in addition to the maximally tolerated volume, beyond which has no benefit or increases the risk of exercise related adverse events. Thus, we recommend that more exercise oncology researchers engage in “dose-finding” trials similar to those in drug development, to better understand the impact of manipulating exercise prescription on muscle mass and function in oncology (Ji et al., 2018; Lee et al., 2021).

## Conclusion

In summary, the field of exercise oncology has made considerable progress within just the last decade. Though considerable gaps remain in our understanding of how exercise may benefit those with muscle dysfunction, these gaps should be viewed as exciting areas of inquiry (Table 2). We hope this review will promote discussion and continued progress in research investigating cancer-related muscle dysfunction.

**TABLE 2:** Overview of suggested considerations for researchers seeking to design studies investigating cancer related muscle dysfunction.

Consideration	Current limitations
Define the condition of interest	There is currently no standardized definition and/or assessment method for various muscle dysfunction conditions such as frailty, sarcopenia, and cancer cachexia
Determine the most appropriate outcome and method of assessment	Debate exists pertaining to the outcome variables of most importance. Namely, muscle mass and function appear to exhibit divergent results in response to exercise interventions. Additionally, methods of functional assessment are not widely agreed upon
Establish the best timepoint along the cancer continuum for intervention	Although exercise prehabilitation may be an effective strategy it is not always feasible. There is limited research on the effectiveness of exercise interventions in those receiving treatment who are also diagnosed with a muscle dysfunction condition/s
Create evidence-based recommendations for how exercise prescription can be configured/implemented to optimize outcomes	Understanding of how the manipulation of key exercise variables (I.e., frequency, intensity, type, and time) applies to conditions where muscle dysfunction is prominent remains limited
